# Clinical results of the *BoneWelding®*Fiji® anchor for the treatment of Stener lesions of the thumb

**DOI:** 10.1007/s00402-020-03625-x

**Published:** 2020-10-10

**Authors:** Tobias Kastenberger, Peter Kaiser, Gernot Schmidle, Kerstin Stock, Stefan Benedikt, Rohit Arora

**Affiliations:** Department of Orthopaedics and Trauma Surgery, Medical University Innsbruck, Anichstr. 35, 6020 Innsbruck, Austria

**Keywords:** Stener, Anchor, Thumb, Ulnar collateral ligament, Repair, Cancellous

## Abstract

**Introduction:**

A new technology (Sportwelding®) was recently presented, which uses ultrasonic energy to meld a resorbable suture anchor at the interface with the host bone. A standardized clinical use was not investigated yet. This study prospectively evaluated the surgical and clinical outcomes of the Fiji Anchor® (Sportwelding®, Schlieren, Switzerland) in the repair of an ulnar collateral ligament lesion of the metacarpophalangeal joint of the thumb.

**Material and methods:**

The range of motion, grip and pinch strength, disability of arm, shoulder and the hand (DASH) and patient rated evaluation (PRWE) score, pain, satisfaction, complications and adverse events were assessed in 24 patients after surgical treatment for an acute displaced rupture or avulsion of the ulnar collateral metacarpophalangeal ligament of the thumb using the Fiji Anchor® after 6, 12 and 52 weeks.

**Results:**

At final follow up, the range of motion of the metacarpophalangeal joint reached almost the contralateral side (49.3° SD 11.7°). Thumb opposition showed a Kapandji score of 9.7 (SD 0.5; range 9–10). Grip strength, the lateral, tip and the three jaw pinch showed nearly similar values compared to the contralateral side (83–101%). Pain was low (0.2 SD 0.7 at rest and 0.6 SD 1.0 during load). The DASH score was 5.0 (SD 7.3) and the PRWE score was 4.1 (SD 9.0). 81% of patients were very satisfied at final follow-up. Two patients were rated unstable during the follow-up period due to a second traumatic event. Three cases experienced difficulties during anchor insertion, whereby incorrect anchor insertion resulted in damage to the suture material; however, this was resolved after additional training.

**Conclusion:**

One advantage of this anchor appears to be its stable fixation in cancellous bone. The surgical treatment of an ulnar collateral ligament lesion of the thumb using the Fiji Anchor® can lead to an excellent clinical outcome with a minor complication rate; however, long-term dangers and the cost effectiveness of the procedure are not known yet.

## Introduction

The traumatic rupture of the ulnar collateral ligament of the metacarpophalangeal joint of the thumb is a common injury to the hand. It is often called Gamekeeper´s or Skier´s thumb. While partial or complete lesions of the ligament with up to 3 mm displacement might be treated conservatively by immobilization, complete ruptures with a ligament displacement of more than 3 mm from its insertion, or a displacement superficial to the adductor aponeurosis (Stener lesion) benefit from surgical treatment [1, 2].

Several different surgical methods have been described using pullout sutures [3–6], suture buttons [3, 6], suture anchors [7–10], interference docking screws [11], condylar shaving with suture anchors [12], arthroscopic repair [13], and suture tape augmentation [14, 15].

A new technology (Sportwelding®) was recently presented, which uses ultrasonic energy to meld a resorbable suture anchor at the interface with the host bone [16]. During the process, the polymer infiltrates the pores of the surrounding cancellous bone in the immediate vicinity of the implant and, following a rapid solidification due to immediate cooling, forms a strong and uniform bond between the implant and bone providing immediate anchor fixation and stability.

This technology was already used in the surgical treatment of hallux valgus [17], in calvarial defects in dogs together with a titanium mesh [18], in oral and maxillofacial surgery [19], in experimental spinal surgery in sheep [20] and can potentially be used in rotator cuff repair [21].

A broad clinical use in particular in hand surgery has not yet been explored or reported. The purpose of this study was to evaluate the surgical and clinical outcome of the SportWelding® Fiji Anchor® (Sportwelding® GmbH, Schlieren, Switzerland) in the direct repair of an acute ulnar collateral ligament rupture. This study was designed to provide evidence on the patient`s outcome and on the safety of this new technology in a clinical setting.

## Patients and methods

Patients enrolled in this prospective case series were above the age of 18 due to receive surgical treatment for a gross ulnar-sided metacarpophalangeal joint instability of the thumb with a 2.3 mm Fiji Anchor® (SportWelding® GmbH, Schlieren, Switzerland) between December 2013 and June 2015. The SportWelding® Fiji Anchor® is an approved (CE-marked) suture anchor made of Poly-l-(d,l)-lactide (70/30) acid (a bioabsorbable polymer based on lactic acid) which is inserted using BoneWelding® technology. Approval to conduct this prospective cohort study was obtained from the ethical review board of the (blinded for review). Informed consent was obtained from all individual participants. This study was conducted according to the ISO 14155 guidelines. No deviation of this international standard was reported or found during the monitoring visits.

Twenty-four patients [6 females, 18 males; mean age 44 years (range 20–71); 23 right-handed, 1 left-handed; 8 smokers (14 (SD 8) cigarettes per day); 2 with diabetes mellitus] were included in this prospective clinical evaluation. Four other patients were excluded as a result of different surgical means being required after inclusion (two mid-substance ligament tear treated with a ligament suture, one sustained a ligament injury of the little finger instead of the metacarpophalangeal joint of the thumb, and one underwent an operation for a palmar metacarpophalangeal instability).

The diagnosis of a displaced ulnar collateral ligament lesion was conducted by clinical assessment and X-ray evaluation. Pain, tenderness, and swelling were present on the ulnar side of the ulnar collateral ligament. Initial dorsovolar and lateral X-rays were either normal or showed a very small displaced avulsed bone fragment. A displaced avulsed fragment was an indication for surgery. In cases where X-ray images were normal, stress images were taken of both, left and right, thumb metacarpophalangeal joints. A side difference of 20° or an absolute value of 35° radial angulation was considered as a displaced ligament indicating surgery.

**Fig. 1:**
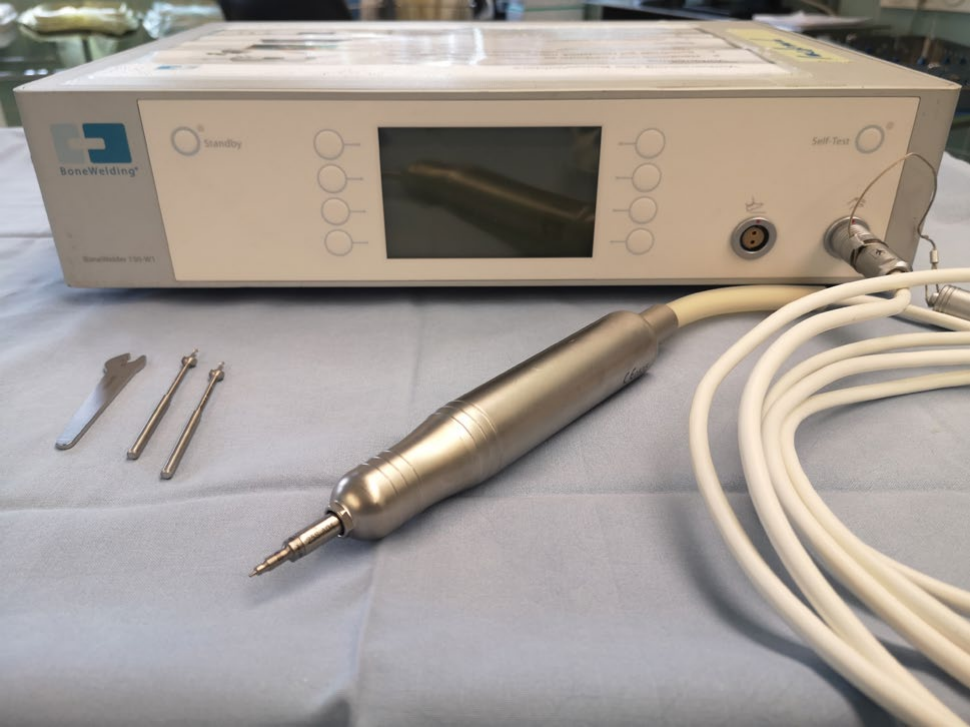
Hand piece, drill bits and BoneWelding® melting machine

**Fig. 2:**
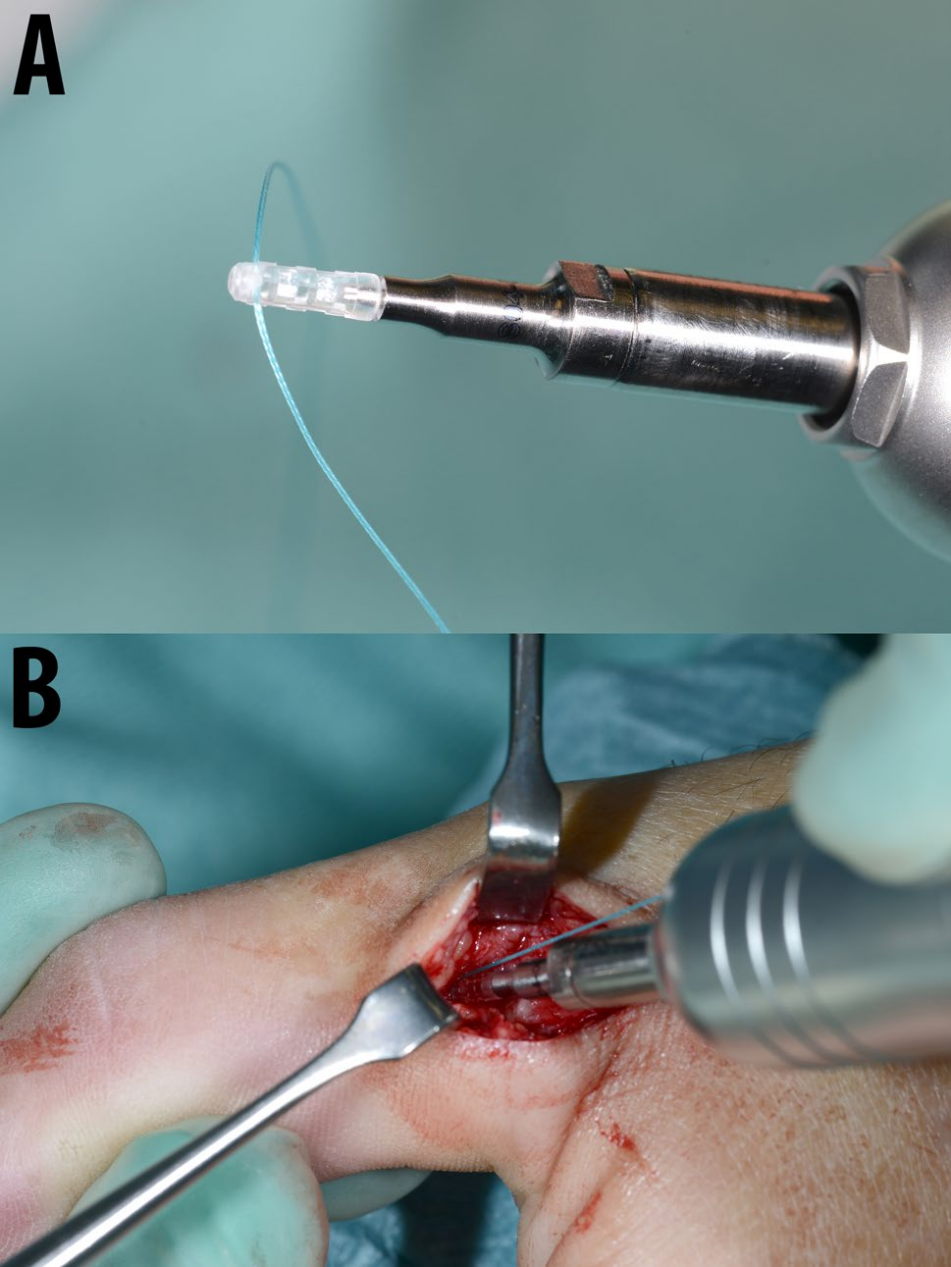
Fiji Anchor® armed with a 3.0 Ethibond suture is sitting on the inserting and melting device (**a**). Intraoperative insertion of the Fiji Anchor® inside the predrilled hole in the base of the proximal phalanx of the thumb during ulnar collateral ligament repair (**b**)

The surgical procedure was conducted via a midline or slightly oblique incision at the site of the ulnar collateral ligament. Blunt dissection was performed to identify and protect the superficial radial nerve, which was protected radially. The adductor aponeurosis was incised longitudinally, and thereafter the joint capsule at its dorsal insertion. All included and evaluated patients presented a rupture of the distal insertion or an avulsion with a Stener lesion. The distal insertion at the base of the proximal phalanx was roughened for ligamentous adhesion and the manufacture’s drill bit was used to drill the anchor hole. The SportWelding® Fiji Anchor® was inserted using BoneWelding® technology in the axis of the drilled hole with a 3.0 non-resorbing suture (Ethibond, Ethicon Inc., Bridgewater, New Jersey, USA). (Figs. 1 and 2) The anchor is inserted into the predrilled hole, while pushing a foot pedal until the anchor is flush with the bone. The anchor melts inside the hole during this procedure. The pedal is pushed until an acoustic signal is heard (roughly 3 s).

The ulnar collateral ligament was sutured to its distal insertion using an U-shaped suture technique. Stability was carefully tested, and the capsule and the adductor aponeurosis were closed before skin closure. All patients were treated with a cast fixation for 5 weeks followed by occupational therapy. Patients were treated by five different specialists in hand surgery. The use of this anchor was only permitted if the doctor had appropriate training. All surgeons, who used this device, underwent a theoretical and practical instruction course by the company and inserted this anchor into a sawbone.

A standardized follow-up protocol was conducted after 6, 12, and 52 weeks. The ulnar collateral ligament stability was tested manually by means of subjective assessment by a doctor. Pain at rest and during load was recorded on the visual analog scale (VAS). Patient satisfaction was recorded with a three option grading system (very satisfied, partly satisfied and not satisfied). The range of motion of the metacarpophalangeal joint was recorded by the use of a finger goniometer. Additionally, thumb opposition was assessed using the Kapandji scale (0–10). Overall grip strength, lateral pinch, tip pinch between the thumb and the index, third, ring and little finger, and the three jaw pinch strength were measured with the “Dynamometer G200” (Biometrics Ltd, Newport, UK). The patients were asked to squeeze the dynamometer three times in a row for grip strength assessment. The mean outcome of the three measurements was used for calculations. The software “E Link SW2111-1196 Version 11.01” (Biometrics Ltd, Newport, UK) was used to assess these parameters. The subjective functional outcome was assessed by the “Disability of the Arm, Shoulder and Hand Score” (DASH) and the “Patient-Rated Wrist Evaluation Score” (PRWE).

Any complications and adverse events (AE) were recorded at each follow-up date.

Results are presented using descriptive statistics which were calculated with Microsoft Excel (Microsoft Corporation, Redmond, Washington, USA).

## Results

Eighteen patients sustained their injury during sporting activities, mainly skiing. Four patients fell from standing height and two did not specify the incident. Seventeen patients (71%) presented a ligamentous lesion and seven patients (29%) a bony avulsion of the ligament. Ten patients (40%) sustained an injury to their dominant hand.

**Fig. 3:**
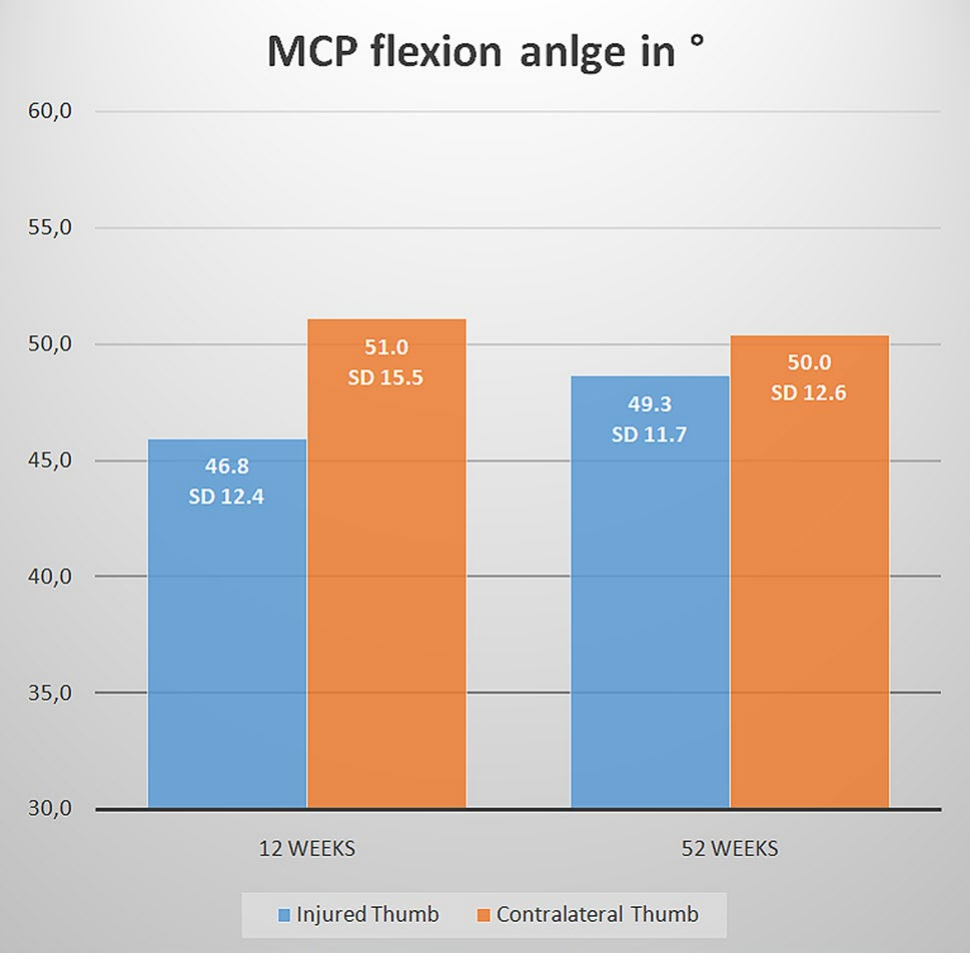
Comparison of metacarpophalangeal (MCP) joint flexion of the thumb after 12 and 52 weeks between the injured and contralateral thumb

Twelve and fifty-two week post-surgery, the thumb showed a very good flexion in the metacarpophalangeal joint, almost reaching the contralateral range of motion (ROM). (Fig. 3) The opposition was also very good with Kapandji scores of 9.1 (SD 0.9; range 7–10) and 9.7 (SD 0.5; range 9–10) after 12 and 52 weeks, respectively. At 52 weeks, two patients (8%) were rated unstable, but were satisfied and refused further surgical treatment.

**Fig. 4:**
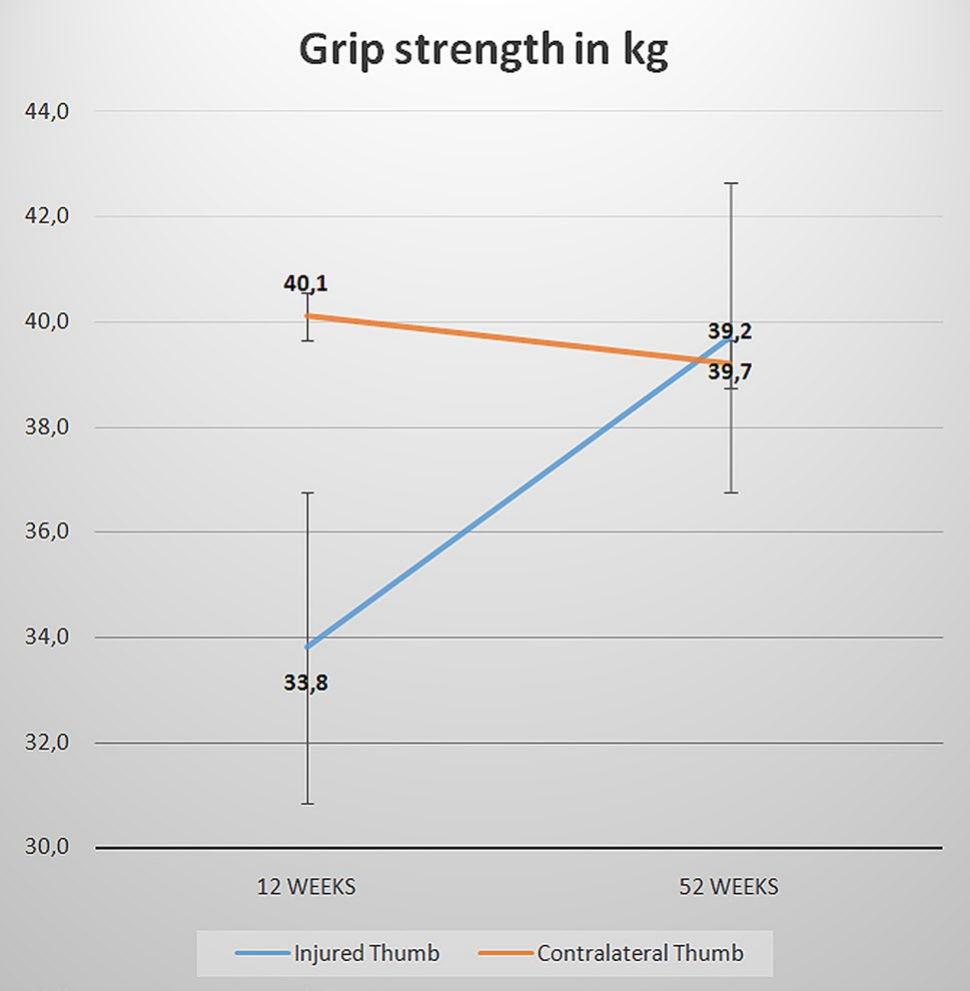
Comparison of the grip strength after 12 and 52 weeks between the injured and contralateral thumb

Grip strength increased during the follow-up period and reached the strength of the contralateral side within 1 year. (Fig. 4).

The lateral pinch, tip pinch, and the three jaw pinch showed nearly similar values compared to the contralateral side 1 year after the surgery (Table 1).

**Table 1:** Lateral, tip and three jaw pinch in kg [mean, standard deviation (SD) and percentage to the contralateral side]

	12 weeks	52 weeks
Lateral pinch	6.7 (SD 2.7)/82%	8.2 (SD 2.9)/99%
Tip pinch D1/D2	4.5 (SD 2.1)/87%	4.9 (SD 2.3)/94%
Tip pinch D1/D3	4.3 (SD 1.7)/86%	4.4 (SD 1.5)/83%
Tip pinch D1/D4	2.8 (SD 1.1)/93%	3.1 (SD 0.9)/94%
Tip pinch D1/D5	2.0 (SD 1.0)/95%	2.0 (SD 0.7)/100%
Three jaw pinch	5.5 (SD 2.3)/79%	6.8 (SD 2.7)/94%

*D* digit

Pain at rest and during load was low during the follow-up period. The subjective functional scores (DASH and PRWE) showed excellent results (Table 2).

**Table 2:** Visual Analog Scale (VAS), Disabilities of Arm, Shoulder and the Hand (DASH) and Patient-Rated Wrist Evaluation (PRWE) Scores

	6 weeks	12 weeks	52 weeks
VAS at rest	0.7 (SD 1.2)	0.3 (SD 0.5)	0.2 (SD 0.7)
VAS during load	–	1.5 (SD 1.6)	0.6 (SD 1.0)
DASH Score	–	9.5 (SD 9.9)	5.0 (SD 7.3)
PRWE Score	–	7.2 (SD 7.2)	4.1 (SD 9.0)

**Fig. 5:**
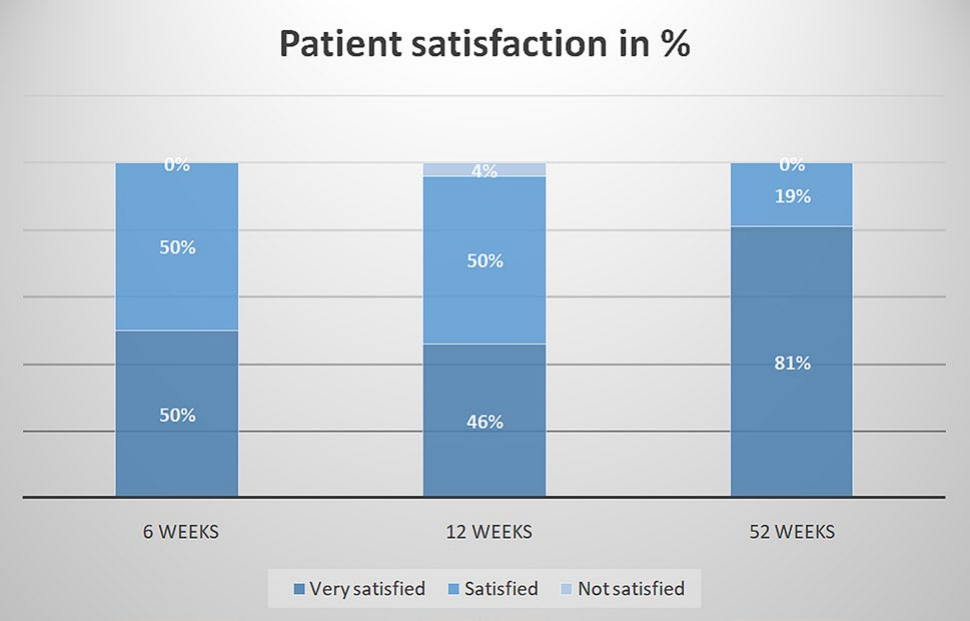
Patient satisfaction rate after 6, 12 and 52 weeks

The patient satisfaction was very high with 81% of patients being very satisfied at final follow-up. (Fig. 5).

The mean surgical time was 39 min (24–86 min).

Complications or AEs were reported in eleven cases. One patient sustained a wrist fracture while playing sports, which was unrelated to the evaluated surgery or anchor. Two patients showed a transient neurapraxia of the superficial radial nerve. One patient presented excessive pain due to an excessively tight cast fixation with relief upon cast change. Three patients presented a subluxation of the metacarpophalangeal joint of the thumb. Two of these cases were trauma related as a result of an additional fall and an injury accidentally inflicted by a baby. The third was rated as pre-existing, as the opposite thumb also showed a subluxation.

Three cases showed difficulties during anchor insertion. They all concerned suture material damage during the insertion process. In two cases the anchors were replaced by another FijiAnchor® in the same position by overdrilling the inserted anchor using the drill bit. The third FijiAnchor® was left in place after removing the damaged sutures with chisels, and the ligament was fixed using a trans-osseous method. No negative consequences for these three patients were reported. These cases were the beginning of the learning curve and were refined with additional and adapted anchor insertion training. In one case the anchor was not inserted completely; however, the minor protruding melted anchor was cut flush to the bone without damaging the suture material and no consequences resulted during the healing process.

No explicit signs of potential device-related risks or hazards were observed in any of the patients.

## Discussion

The most important finding of this study was that surgical ligamentous repair of the ulnar collateral ligament of the metacarpophalangeal joint using the Fiji Anchor® leads to excellent clinical results with a minor complication rate.

These good clinical results are comparable to previously published reports using various different kinds of fixation techniques for this type of injury [5, 13, 15, 22–26]. A detailed compilation of the results from the current study in comparison with previous reports is shown in Table 3. In this study thumb metacarpophalangeal joint flexion values are quite similar to previous reports with 99% of the contralateral side irrespective of the treatment method in the other studies. Grip strength and lateral pinch strength fall into a similar range as previously reported, and similarly, pain was rarely an issue, with most patients pain free, and only mild pain recorded on occasion. Lee et al. describes the QuickDASH scores of their patients, which were slightly higher than our DASH scores; however, all scores are relatively low considering the absolute value. Our results, and the results of previous reports, as shown in Table 3, demonstrate that the treatment of an acute ulnar collateral ligament rupture leads to very good results, irrespective of the surgical treatment choice.

**Table 3:** Overview of outcome paramaters regarding different surgical treatment techniques in the literature

Author	Year of publication	Patient number	Mean follow-up	Treatment	Outcome
Katolik et al	2008	30 per group	29 months	Comparison between pull-out suture (PS) and bone anchor (BA)	(PS/BA) ROM 97%/87% of contralateral side MP and IP joint Pinch strength 101%/No diff Grip strength No diff./No diff Tourniquet time 28/43 min Complications 27%/7% Persistent erythema suggestive of infection *n* = 3/*n* = 2 Prolonged paresthesia *n* = 5/*n* = 0 Satisfaction 4.9/4.5
Jackson and McQueen	1994	19	17 months	Retrospective review of the results of an acute surgical repair	ROM No limitations of thumb movement Pinch strength No significant diff. between palmar and lateral pinch grip pre- and post-injury Strength No weakness *n* = 14, mild weakness *n* = 3, significant weakness *n* = 2 Stability All stable Pain None *n* = 13 Mild *n* = 6 Complications Minor discomfort *n* = 6
Bostok et al	1993	14	44 months	Ligament repair *n* = 8 K-wire fixation of ligament avulsion *n* = 4 Drill hole reattachment *n* = 1 Anchor repair *n* = 1	ROM 51° (31–64°)/86% No overall loss of movement in comparison injured to uninjured thumb Strength Loss of grip without interference of daily activity 4/14 Complications Discomfort *n* = 6
Ryu and Fagan	1995	8	39 months	Arthroscopic ligament repair	ROM 51° MP joint 102° IP joint Tip pinch strength 13 lbs Pinch strength 19 lbs Grip strength 98 lbs Surgical time 47 min Complications 1 pin infection
Downey et al	1995	11	42 months	Pullout suture repair	ROM 51° MP joint/69% of contralateral side 102° IP joint/90% of contralateral side Grip strength 32 kg/95% of contralateral side No significant side difference Key pinch 8 kg/92% of contralateral side No significant side difference Stability 14° ulnar laxity/92% of contralateral side (injured thumb more stable VAS 2 (1–4)
Kara et al	2019	12	22 months	Anchor repair	ROM Same level as the contralateral side Grip strength 94% of contralateral side Pinch strength 92% of contralateral side No complications
Lee et al	2020	13	Min. 12 months	Ligament repair with suture tape augmentation	ROM 58° MP joint 71° IP joint Grip strength 102% of contralateral side Pinch strength 84% of contralateral side QuickDASH 12 (disability module)/0 (sport module)/17 (work module) Four reported stiffness All stable with firm endpoint No complications
Lane	1991	32	47 months	Pullout suture repair + K-wire transfixation *n* = 7 Suture to the tendineous insertion of the adductor pollicis *n* = 22? K-wire fixation of ligament avulsion *n* = 3?	ROM 82% of total active MP + IP ROM Strength, pain and stability corrected Complications Broken pull-out suture *n* = 1 Ligamentous rerupture after new trauma *n* = 1

Three months post-surgery, the range of motion, grip strength, lateral pinch, tip pinch and three jaw pinch were 79–95% compared with the contralateral side. Within 1 year, these measures were almost 100%. All patients were treated with a static immobilization for 5 weeks; however, early motion treatment could potentially lead to even faster results, at least in the short term [5, 27, 28].

Considering that all studies report a good clinical outcome, the means of fixation play a minor role. Undisplaced ligament lesions can be successfully treated non-surgically [3, 4, 29]; however, displaced ligaments have better clinical outcomes in surgically treated patients, the reason being that placing the ligament into its anatomical insertion is likely sufficient for successful healing independent of any fixation methods. Speculatively speaking, it is possible that no fixation method is necessary at all if the ligament is turned from its fold back position (Stener lesion) to its original distal insertion and only be laid underneath the adductor aponeurosis. However, this is yet to be elucidated.

Suture anchor fixation has been shown to lead to a better outcome than a pullout suture [5]. Suture anchors are, however, not always suitable. Displaced ligamentous avulsions need k-wire, screw, or hook plate fixation as conservative treatment has frequently yielded poorer clinical results [30, 31]. However, there are cases with small or multi-fragmentary avulsions for which these fixation methods are not suitable. The Fiji Anchor® shows one potential advantage in such cases. While other anchors need cortical support as a resistance for stable anchor fixation, this anchor can melt into cancellous bone and lead to a strong and stable fixation. It was shown biomechanically, that this anchor withstood significantly higher pullout forces than a hard or soft anchor in avulsion fractures providing sufficient anchorage in trabecular bone for ulnar collateral ligament repair [32].

Although complications using this anchor did occur in the present study, many were directly related to the surgery or treatment, such as transient neurapraxia of the superficial radial nerve or pain due to a cast fixation, rather than specifically caused by the fixation method itself. Others were due to unfortunate events such as acute falls or accidents caused by children. The main complication involving the implant itself, was damage to suture material during insertion of the anchor. This was most likely due to the lack of experience with this surgical procedure. Typically, surgeons place suture anchors exerting a certain force to push the anchor deep under the cortical bone. This is because the vast majority of anchors on the market hold on to the subcortcial bone with barbs or by other means. Using the BoneWelding® technology, the polymer interdigitates with the surrounding bone over the entire anchor surface without the need of an intact cortical bone. The anchor is inserted without impact forces, and only until it is flush to the bone surface. If the process does not stop at this point, there is a certain risk, especially when the anchor is not inserted on the exact axis of the drill hole, that the activated sonotrode may damage the suture material if it is firmly pressed against hard cortical bone for a long enough time. After the third occurrence of suture damage during insertion, surgeon’s attention was directed to stop the insertion process at the moment the anchor is implanted flush with the bone surface, as well as inserting the anchor on the exact axis of the drilled hole. After this additional training and the experience gained with the procedure, no further suture damage occurred during insertion. Regarding these complications, it has to be emphasized that the introduction of the anchor is accompanied by a small learning curve. However, after understanding the nature of this complication, it can be avoided safely.

In cases of suture damage or a protruding anchor, the inserted anchor can be overdrilled and a new Fiji Anchor® can be inserted and melted into the hole with a stable fixation.

No iatrogenic injuries were caused by the tested anchor and no anchor-related failures were observed during the surgery. Based on these results, the safety of using the Fiji Anchor® for refixations and reconstructions of ligaments in the hand is good.

As the anchor proclaims to be bioabsorble, no material should remain after resorption. This may potentially lead to osteolysis, failure of fixation, reoccurrence of instability, pain or other complications. The follow up study period was not long enough to report on such complications, because according to the manufacturer, resorption takes places after 2 or more years. Currently, there are no published studies demonstrating the duration at what point the anchor resolves, and if there are any radiologic consequences such as potential osteolysis.

This was a limitation in the current study, that radiological evaluation was not conducted. Moreover, the follow-up period was also too short to evaluate further mid- and long-term complications like early osteoarthritis. Then again, if existing osteoarthritis pain did appear to be of little relevance as the patients did not suffer any significant pain during the follow-up period. Furthermore, there was no control group, where different fixation systems were used, and therefore, superiority over other methods cannot be deduced. Nevertheless, as previously stated, the clinical functional outcome does not appear to be related to any surgical treatment technique. Finally, patients were not included in a sequential order. Patients treated by another means of fixation during the inclusion period were not included in the study. At last, the use of this new anchor needs a special apparatus and inserting device that needs to be purchased. This is contrary to other means of fixation which don´t need other machines. Regarding the economic side, costs for any fixation device or any comparisons cannot be depicted; however, an anchorless fixation will always be cheaper than fixations using external material. Future economic calculations must compare the benefits and the costs of this anchor.

## Conclusion

The surgical treatment of an ulnar collateral ligament lesion of the thumb leads to an excellent clinical outcome with a minor complication rate using the Fiji Anchor®. The advantage of this anchor is its stable fixation in cancellous bone in the absence of an intact cortex. However, the insertion of this anchor shows a moderate learning curve, and long-term risks and the cost-effectiveness are unknown.
